# Effects of a Moisturizer and Emollient on the Stratum Corneum Assessed by Confocal Raman Spectroscopy in Patients With and Without Atopic Dermatitis: An Assessor‐Blinded Prospective Cohort Study

**DOI:** 10.1002/hsr2.72671

**Published:** 2026-06-19

**Authors:** Risa Fukuda, Rena Hashimoto, Kyongsun Pak, Hazuki Yasuda, Naoko Mochimaru, Yukio Matsumoto, Megumi Kiuchi, Shiho Uematsu, Kazue Yoshida

**Affiliations:** ^1^ National Center for Child Health and Development Division of Dermatology Tokyo Japan; ^2^ Department of Data Management, Center of Clinical Research and Development, National Center for Child Health and Development Division of Biostatistics Tokyo Japan; ^3^ Department of Product Development Pigeon Corporation Ibaraki Japan; ^4^ Department of Product Development Pigeon Home Products Corporation Shizuoka Japan

**Keywords:** atopic dermatitis, confocal Raman spectroscopy, emollient, moisturizer, skin physiology, structure

## Abstract

**Background and Aims:**

Atopic dermatitis (AD) is characterized by impaired skin barrier function and reduced moisturizing factors. Moisturizers and emollients restore the barrier through distinct mechanisms, but their longitudinal molecular effects on the stratum corneum (SC) are poorly characterized, particularly in barrier‐deficient skin. We assessed the time‐dependent effects of a cream‐type moisturizer (CM) and petroleum jelly (PJ) on SC molecular composition in adults with and without AD using confocal Raman spectroscopy (CRS).

**Methods:**

In this assessor‐blinded prospective cohort study (November 2019–February 2020), 12 adults without AD and 12 with AD applied 0.5 g each of CM and PJ to randomly assigned right or left forearms once daily for 28 (+ 7) days. SC water content, natural moisturizing factor (NMF), ceramide, cholesterol, and lactic acid were measured by CRS at 0, 14, and 28 days; transepidermal water loss (TEWL) and SC hydration were also assessed. Welch's *t*‐tests with Bonferroni correction compared days 0–14 and 0–28 (two‐sided, 5% significance).

**Results:**

Twenty‐three participants (non‐AD, *n* = 12, 42.8 ± 8.0 years; AD, *n* = 11, 41.5 ± 9.1 years) completed the study. Ceramide increased over time, mainly in the upper SC: CM raised ceramide significantly in both groups, whereas PJ significantly increased ceramide across all layers only in the non‐AD group. CM significantly increased lactic acid across all layers in non‐AD participants and in upper layers of AD participants, while PJ had no effect. SC hydration rose with both agents in non‐AD participants but only with CM in AD participants. TEWL showed no significant changes.

**Conclusion:**

Moisturizers and emollients exerted distinct effects on SC components. Moisturizers were superior, increasing ceramide and lactic acid in the upper SC of patients with AD, and appear better suited for managing skin barrier dysfunction.

## 1 Introduction

Atopic dermatitis (AD) is characterized by impaired skin barrier function and reduced moisturizing factors, leading to dry skin. Moisturizers and emollients increase water content in the stratum corneum (SC) and restore skin barrier function by different mechanisms. Moisturizers contain humectants, which attract water [1], whereas emollients form an oily layer to prevent water loss and replenish SC moisture from deeper skin layers [2].

Their efficacy varies by formulation. Petrolatum is a well‐established occlusive agent that enhances SC hydration and barrier recovery [1]. The lipid composition and microstructure of topical formulations influence occlusivity and barrier repair, with fatty acids enhancing ceramide synthesis and unsaponifiable fractions providing anti‐inflammatory and barrier repair effects [3, 4, 5].

Recent studies have evaluated topical skincare products using minimally invasive methods (e.g., tape stripping and solvent‐based analysis) [6, 7, 8]. Research employing confocal Raman spectroscopy (CRS), a noninvasive technique for detailed SC molecular analysis, remains limited [9]. This is the first study to investigate the longitudinal effects of topical agents on SC molecular changes both with and without skin barrier dysfunction.

## 2 Methods

In total, 12 adults without AD and 12 adults with dermatologist‐diagnosed or self‐reported AD (the non‐AD and AD groups, respectively) were recruited between November 2019 and February 2020. Patients who had used oral or topical steroids, immunosuppressants, or tacrolimus on the forearms within 1 month were excluded.

Participants in each group applied 0.5 g of a cream‐type moisturizer (CM) (Pigeon Baby Cream F, Pigeon Corp., Tokyo, Japan) and petroleum jelly (PJ) (Pigeon Vaseline P, Pigeon Corp.) on the right or left forearm once daily after bathing for 28 (+7) days. The CM contained botanical ingredients including sagarame and orange peel extract (Supporting Information S1: Table S1), whereas the PJ consisted of pure petrolatum. Adherence was assessed using questionnaires at 28 days. The participants were randomly assigned to a treatment arm (1:1 ratio) (Table 1); they were not blinded, but measurement staff were.

**Table 1:** Characteristics of groups with and without a history of atopic dermatitis (AD).

Factors	Classes	Non‐AD (*n* = 12) *n* (%)	AD (*n* = 11) *n* (%)
Age (y. mean ± SD)	—	42.8 ± 8.0	41.5 ± 9.1
Sex	Male n (%)	6 (50.0%)	6 (54.5%)
	Female n (%)	6 (50.0%)	5 (45.5%)
Application site	CM (left)/PJ (right)	7 (58.3%)	4 (36.4%)
	CM (right)/PJ (left)	5 (41.7%)	7 (63.6%)
Family history of AD	Yes	1 (8.3%)	3 (27.3%)
	No	11 (91.7%)	8 (72.7%)
Bath frequency	Once daily	12 (100.0%)	10 (90.9%)
	Twice or more daily	0 (0.0%)	1 (9.1%)
Wash with soap in bath	Yes	10 (83.3%)	11 (100.0%)
	No	2 (16.7%)	0 (0.0%)
Body washing	Hand	7 (58.3%)	7 (63.6%)
	Towel or sponge	3 (25.0%)	3 (27.3%)
	Others	1 (8.3%)	1 (9.1%)
	Unknown	1 (8.3%)	0 (0.0%)
Moisturizer use	Yes	5 (41.7%)	1 (9.1%)
	No	7 (58.3%)	10 (90.9%)

Abbreviations: AD, atopic dermatitis; CM, cream‐type moisturizer; PJ, petroleum jelly; SD, standard deviation.

The SC molecular composition was measured before application and at 14 (+7) and 28 (+7) days. Questionnaires were administered at 28 days. SC parameters assessed included water content, natural moisturizing factor (NMF), total ceramide, cholesterol, lactic acid, urocanic acid (pH 4 and 8), urea, and keratin, which were measured using a CRS model 3510 (River Diagnostics BV, Rotterdam, The Netherlands). Each measurement was performed five times, and mean values were analyzed. Transepidermal water loss (TEWL) and SC hydration were evaluated using a Tewameter (TM300; Courage & Khazaka, Köln, Germany) and a Corneometer (CM825; Courage & Khazaka) [10].

All measurements were performed at 4‐μm depth intervals on the inner forearms at 22°C ± 2°C and 50% ± 10% humidity. Ointment application to the measurement sites was prohibited on assessment days. Welch's *t*‐test was used to compare days 0–14 and 0–28, with Bonferroni correction for multiple tests. Continuous variables are presented as means with 95% confidence intervals, using a two‐sided 5% significance level. All analyses were performed using R version 4.1.2 (R Foundation, Vienna, Austria) and GraphPad Prism version 10.0 (GraphPad Software, San Diego, CA, USA).

## 3 Results

The final analysis included 12 patients without AD (age: 42.8 ± 8.0 years) and 11 patients with AD (age: 41.5 ± 9.1 years); 1 patient with AD was excluded because of a rash (Table 1). Application adherence was high in both groups, with comparable distributions of family history of allergies or bathing frequency (Table 1).

**Figure 1:**
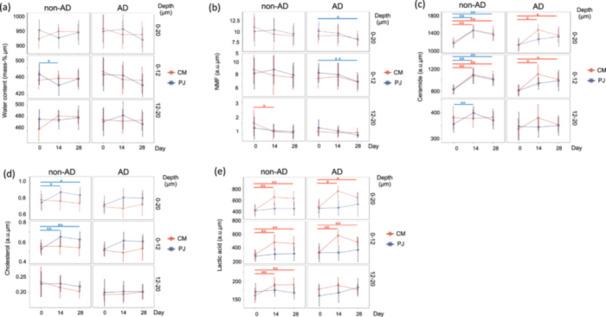
Changes in the molecular composition of the stratum corneum at 0, 14, and 28 days with CM and PJ in patients with and without AD, measured by confocal Raman spectroscopy. The mean area under the curve (0–20, 0–12, and 12–20 μm) is shown for (a) water content, (b) NMF, (c) ceramide, (d) cholesterol, and (e) lactic acid. CM is represented by circles with a red line, and PJ is represented by squares with a blue line (*n* = 23). Error bars indicate 95% confidence intervals. *p < 0.05, **p < 0.01. AD, atopic dermatitis; CM, cream‐type moisturizer; NMF, natural moisturizing factor; PJ, petroleum jelly.

Before application, water content and lactic acid were higher in the AD group, whereas NMF, ceramide, cholesterol, and SC hydration were higher in the non‐AD group (Supporting Information S1: Tables S2 and S3). In the non‐AD group, water content slightly increased overall but decreased significantly with PJ at day 14; the AD group showed a slight overall decrease (Supporting Information S1: Figure 1a and Supporting Information S1: Table S2a). NMF levels trended downward, with significant decreases at day 14 in the deep layer of the non‐AD group with CM and at day 28 in the upper layer of the AD group with PJ (Figure 1b and Supporting Information S1: Table S2b).

Ceramide increased over time, particularly in the upper SC. CM significantly increased ceramide in both groups, while PJ significantly increased it across all layers in the non‐AD group (Figure 1c and Supporting Information S1: Table S2c). Cholesterol showed an increasing trend, with a significant rise in the upper SC of the non‐AD group with PJ (Figure 1d and Supporting Information S1: Table S2d). Lactic acid increased significantly with CM over time across all layers in the non‐AD group and in the upper layers in the AD group. PJ did not significantly affect lactic acid (Figure 1e). Other parameters showed no significant changes associated with treatment or group over time.

SC hydration increased over time with both CM and PJ in the non‐AD group, but only with CM in the AD group (Supporting Information S1: Figure S1a and Table S3a). TEWL showed no significant differences between groups or treatments (Supporting Information S1: Figure S1b and Table S3b).

## 4 Discussion

In this study, both CM and PJ induced molecular changes, with ceramide and cholesterol increasing mainly in the upper SC. Both CM and PJ induced molecular changes, with ceramide and cholesterol increasing mainly in the upper SC. CM improved SC components in both groups and was more effective than PJ, especially in AD.

Baseline values showed no significant group differences (Supporting Information S1: Table S2), likely because of dry skin in non‐AD participants and minimal dryness in AD patients. This overlap may have influenced the observed changes. CM consistently improved SC components, outperforming PJ in AD and aiding barrier dysfunction even when mild.

CM increased ceramide in both groups, consistent with prior studies [8]. It also increased sweat‐derived lactic acid [11], which in turn enhanced barrier function through water retention [12, 13, 14]. Reduced sweating in AD contributes to dry skin, and the increase in lactic acid is consistent with reports that moisturizers promoting sweating improve hydration [7]. PJ increased ceramide and cholesterol only in the non‐AD group. Petrolatum, which penetrates approximately 35% of the SC (approximately 7 μm) [9], enhances water content in the upper SC via its occlusive effect and improves lipid organization in healthy skin [15], explaining lipid increases in the non‐AD group. The lack of effect in AD may reflect lower baseline lipid levels and loss of emollient during bathing. These findings may also reflect the effects of emollient lipid components, including fatty acids and unsaponifiable fractions, on ceramide metabolism and barrier repair. Water content and SC hydration are often negatively correlated [16], with corneometers assessing the upper SC and CRS deeper layers. Conflicting hydration and water content in the non‐AD group imply that PJ primarily affected the upper SC.

Plant‐based moisturizers can enhance barrier function and reduce inflammation in AD [17]. Sagarame (*Eisenia arborea*) increases filaggrin expression [18], while hesperidin in mandarin orange peel extract (*Citrus reticulata*) upregulates filaggrin, lipid synthase, and lipid transport proteins [19, 20]. These may have contributed to ceramide and cholesterol increases, although NMF was unaffected. Further study is needed.

The small sample size may have limited the statistical power, and botanical ingredients could have influenced SC components, adding a confounder. Still, this is the first study using CRS to assess time‐dependent effects of a moisturizer and an emollient on SC components with and without barrier abnormalities. CRS offers robust noninvasive analysis [9], making the findings relevant for clinicians and industry.

## 5 Conclusions

Moisturizers and emollients showed distinct effects on SC components. Moisturizers were superior, increasing ceramide and lactic acid levels in the upper SC of patients with AD, and they appeared better suited for managing skin barrier dysfunction.

## Author Contributions

**Risa Fukuda:** writing – original draft preparation, visualization, formal analysis. **Rena Hashimoto:** writing – original draft preparation. **Kyongsun Pak:** software, data curation, visualization, formal analysis. **Hazuki Yasuda:** conceptualization, methodology, investigation. **Naoko Mochimaru** and **Yukio Matsumoto:** investigation. **Megumi Kiuchi** and **Shiho Uematsu:** conceptualization, methodology, investigation, resources, writing – reviewing and editing. **Kazue Yoshida:** conceptualization, methodology, investigation, writing – reviewing and editing, project administration, supervision. All authors have read and approved the final version of the manuscript. **Kazue Yoshida** had full access to all of the data in this study and takes complete responsibility for the integrity of the data and the accuracy of the data analysis.

## Ethics Statement

This study was approved by the Institutional Ethics Committees of the National Center for Child Health and Development (approval number, 2292). The study was conducted after written informed consent was obtained from each participant's family members.

## Conflicts of Interest

Kazue Yoshida received funding from the Pigeon Corporation, Maruho Company, Ltd., and Natural Science Company, Ltd. Megumi Kiuchi is employed by the Pigeon Corporation. Shiho Uematsu is employed by the Pigeon Home Products Corporation.

## Transparency Statement

Kazue Yoshida affirms that this manuscript is an honest, accurate, and transparent account of the study being reported; that no important aspects of the study have been omitted; and that any discrepancies from the study as planned (and, if relevant, registered) have been explained.

## Supporting information

Supporting File

## Data Availability

The data that support the findings of this study are available on request from the corresponding author. The data are not publicly available due to privacy or ethical restrictions.
