# Supplementary material for: Effects of a Moisturizer and Emollient on the Stratum Corneum Assessed by Confocal Raman Spectroscopy in Patients With and Without Atopic Dermatitis: An Assessor‐Blinded Prospective Cohort Study
Source: Health Sci Rep. 2026 Jun 19;9(6):e72671. doi: 10.1002/hsr2.72671 (PMC13282258; doi:10.1002/hsr2.72671)
Supplement: Supplementary file 1 — Supporting File [file HSR2-9-e72671-s001.pptx]

## Slide 1
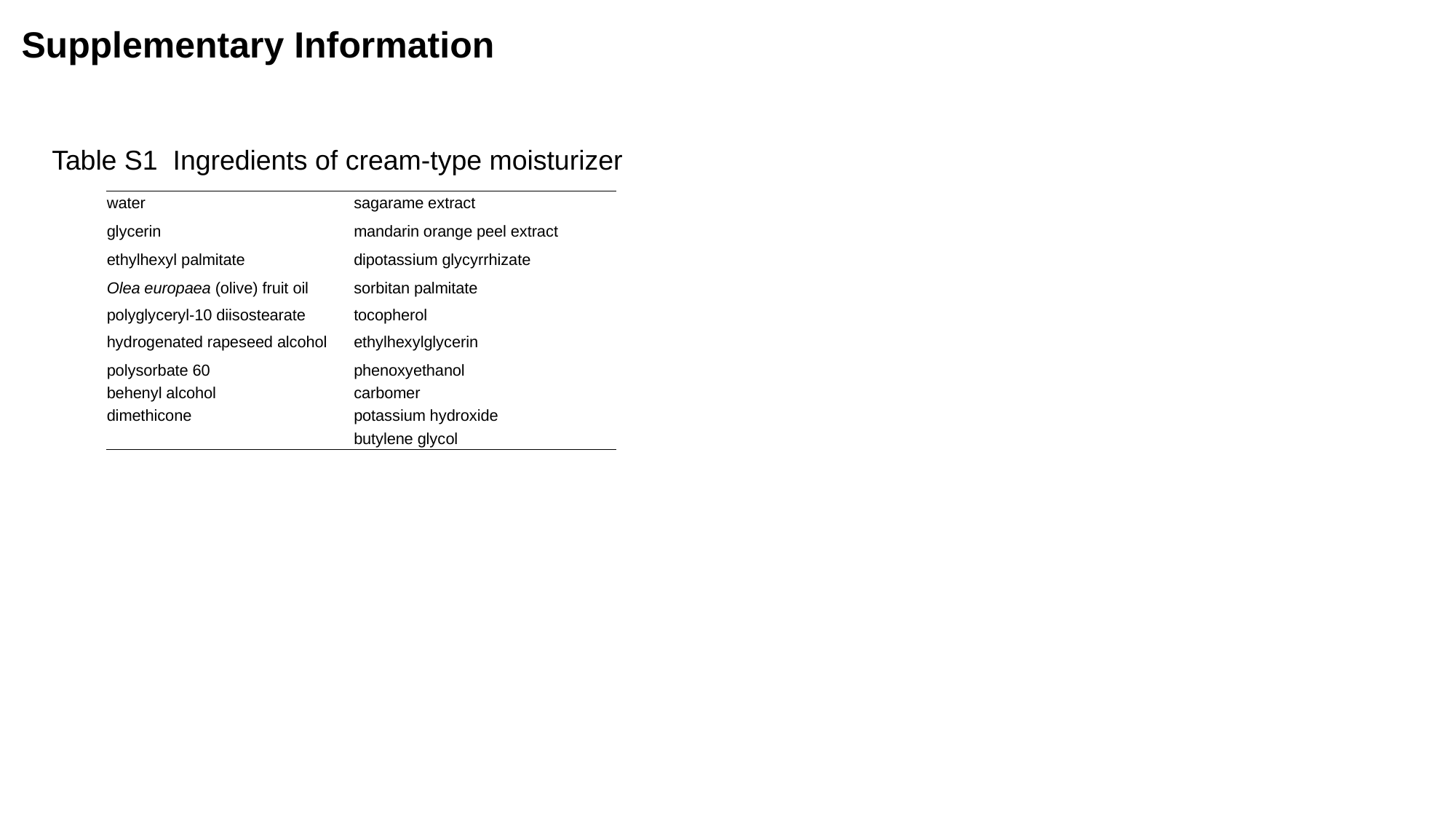

Supplementary Information
Table S1 Ingredients of cream-type moisturizer
| water | sagarame extract |
| --- | --- |
| glycerin | mandarin orange peel extract |
| ethylhexyl palmitate | dipotassium glycyrrhizate |
| Olea europaea (olive) fruit oil | sorbitan palmitate |
| polyglyceryl-10 diisostearate | tocopherol |
| hydrogenated rapeseed alcohol | ethylhexylglycerin |
| polysorbate 60 | phenoxyethanol |
| behenyl alcohol | carbomer |
| dimethicone | potassium hydroxide |
| | butylene glycol |

## Slide 2
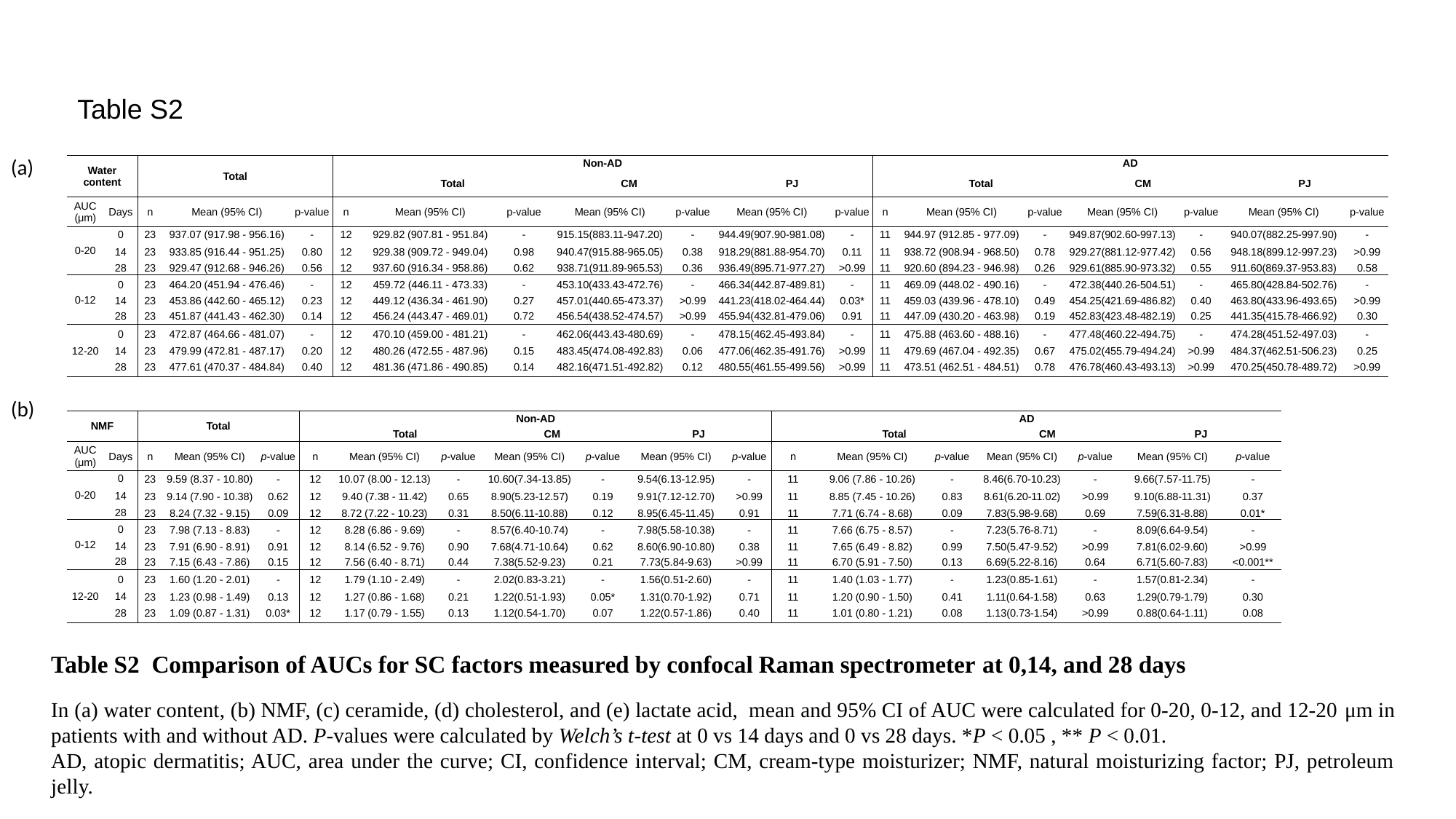

Table S2
(a)
| Water content | | Total | | | Non-AD | | | | | | | AD | | | | | | |
| --- | --- | --- | --- | --- | --- | --- | --- | --- | --- | --- | --- | --- | --- | --- | --- | --- | --- | --- |
| | | | | | | Total | | CM | | PJ | | | Total | | CM | | PJ | |
| AUC (μm) | Days | n | Mean (95% CI) | p-value | n | Mean (95% CI) | p-value | Mean (95% CI) | p-value | Mean (95% CI) | p-value | n | Mean (95% CI) | p-value | Mean (95% CI) | p-value | Mean (95% CI) | p-value |
| 0-20 | 0 | 23 | 937.07 (917.98 - 956.16) | - | 12 | 929.82 (907.81 - 951.84) | - | 915.15(883.11-947.20) | - | 944.49(907.90-981.08) | - | 11 | 944.97 (912.85 - 977.09) | - | 949.87(902.60-997.13) | - | 940.07(882.25-997.90) | - |
| | 14 | 23 | 933.85 (916.44 - 951.25) | 0.80 | 12 | 929.38 (909.72 - 949.04) | 0.98 | 940.47(915.88-965.05) | 0.38 | 918.29(881.88-954.70) | 0.11 | 11 | 938.72 (908.94 - 968.50) | 0.78 | 929.27(881.12-977.42) | 0.56 | 948.18(899.12-997.23) | >0.99 |
| | 28 | 23 | 929.47 (912.68 - 946.26) | 0.56 | 12 | 937.60 (916.34 - 958.86) | 0.62 | 938.71(911.89-965.53) | 0.36 | 936.49(895.71-977.27) | >0.99 | 11 | 920.60 (894.23 - 946.98) | 0.26 | 929.61(885.90-973.32) | 0.55 | 911.60(869.37-953.83) | 0.58 |
| 0-12 | 0 | 23 | 464.20 (451.94 - 476.46) | - | 12 | 459.72 (446.11 - 473.33) | - | 453.10(433.43-472.76) | - | 466.34(442.87-489.81) | - | 11 | 469.09 (448.02 - 490.16) | - | 472.38(440.26-504.51) | - | 465.80(428.84-502.76) | - |
| | 14 | 23 | 453.86 (442.60 - 465.12) | 0.23 | 12 | 449.12 (436.34 - 461.90) | 0.27 | 457.01(440.65-473.37) | >0.99 | 441.23(418.02-464.44) | 0.03\* | 11 | 459.03 (439.96 - 478.10) | 0.49 | 454.25(421.69-486.82) | 0.40 | 463.80(433.96-493.65) | >0.99 |
| | 28 | 23 | 451.87 (441.43 - 462.30) | 0.14 | 12 | 456.24 (443.47 - 469.01) | 0.72 | 456.54(438.52-474.57) | >0.99 | 455.94(432.81-479.06) | 0.91 | 11 | 447.09 (430.20 - 463.98) | 0.19 | 452.83(423.48-482.19) | 0.25 | 441.35(415.78-466.92) | 0.30 |
| 12-20 | 0 | 23 | 472.87 (464.66 - 481.07) | - | 12 | 470.10 (459.00 - 481.21) | - | 462.06(443.43-480.69) | - | 478.15(462.45-493.84) | - | 11 | 475.88 (463.60 - 488.16) | - | 477.48(460.22-494.75) | - | 474.28(451.52-497.03) | - |
| | 14 | 23 | 479.99 (472.81 - 487.17) | 0.20 | 12 | 480.26 (472.55 - 487.96) | 0.15 | 483.45(474.08-492.83) | 0.06 | 477.06(462.35-491.76) | >0.99 | 11 | 479.69 (467.04 - 492.35) | 0.67 | 475.02(455.79-494.24) | >0.99 | 484.37(462.51-506.23) | 0.25 |
| | 28 | 23 | 477.61 (470.37 - 484.84) | 0.40 | 12 | 481.36 (471.86 - 490.85) | 0.14 | 482.16(471.51-492.82) | 0.12 | 480.55(461.55-499.56) | >0.99 | 11 | 473.51 (462.51 - 484.51) | 0.78 | 476.78(460.43-493.13) | >0.99 | 470.25(450.78-489.72) | >0.99 |
(b)
| NMF | | Total | | | Non-AD | | | | | | | AD | | | | | | |
| --- | --- | --- | --- | --- | --- | --- | --- | --- | --- | --- | --- | --- | --- | --- | --- | --- | --- | --- |
| | | | | | | Total | | CM | | PJ | | | Total | | CM | | PJ | |
| AUC (μm) | Days | n | Mean (95% CI) | p-value | n | Mean (95% CI) | p-value | Mean (95% CI) | p-value | Mean (95% CI) | p-value | n | Mean (95% CI) | p-value | Mean (95% CI) | p-value | Mean (95% CI) | p-value |
| 0-20 | 0 | 23 | 9.59 (8.37 - 10.80) | - | 12 | 10.07 (8.00 - 12.13) | - | 10.60(7.34-13.85) | - | 9.54(6.13-12.95) | - | 11 | 9.06 (7.86 - 10.26) | - | 8.46(6.70-10.23) | - | 9.66(7.57-11.75) | - |
| | 14 | 23 | 9.14 (7.90 - 10.38) | 0.62 | 12 | 9.40 (7.38 - 11.42) | 0.65 | 8.90(5.23-12.57) | 0.19 | 9.91(7.12-12.70) | >0.99 | 11 | 8.85 (7.45 - 10.26) | 0.83 | 8.61(6.20-11.02) | >0.99 | 9.10(6.88-11.31) | 0.37 |
| | 28 | 23 | 8.24 (7.32 - 9.15) | 0.09 | 12 | 8.72 (7.22 - 10.23) | 0.31 | 8.50(6.11-10.88) | 0.12 | 8.95(6.45-11.45) | 0.91 | 11 | 7.71 (6.74 - 8.68) | 0.09 | 7.83(5.98-9.68) | 0.69 | 7.59(6.31-8.88) | 0.01\* |
| 0-12 | 0 | 23 | 7.98 (7.13 - 8.83) | - | 12 | 8.28 (6.86 - 9.69) | - | 8.57(6.40-10.74) | - | 7.98(5.58-10.38) | - | 11 | 7.66 (6.75 - 8.57) | - | 7.23(5.76-8.71) | - | 8.09(6.64-9.54) | - |
| | 14 | 23 | 7.91 (6.90 - 8.91) | 0.91 | 12 | 8.14 (6.52 - 9.76) | 0.90 | 7.68(4.71-10.64) | 0.62 | 8.60(6.90-10.80) | 0.38 | 11 | 7.65 (6.49 - 8.82) | 0.99 | 7.50(5.47-9.52) | >0.99 | 7.81(6.02-9.60) | >0.99 |
| | 28 | 23 | 7.15 (6.43 - 7.86) | 0.15 | 12 | 7.56 (6.40 - 8.71) | 0.44 | 7.38(5.52-9.23) | 0.21 | 7.73(5.84-9.63) | >0.99 | 11 | 6.70 (5.91 - 7.50) | 0.13 | 6.69(5.22-8.16) | 0.64 | 6.71(5.60-7.83) | <0.001\*\* |
| 12-20 | 0 | 23 | 1.60 (1.20 - 2.01) | - | 12 | 1.79 (1.10 - 2.49) | - | 2.02(0.83-3.21) | - | 1.56(0.51-2.60) | - | 11 | 1.40 (1.03 - 1.77) | - | 1.23(0.85-1.61) | - | 1.57(0.81-2.34) | - |
| | 14 | 23 | 1.23 (0.98 - 1.49) | 0.13 | 12 | 1.27 (0.86 - 1.68) | 0.21 | 1.22(0.51-1.93) | 0.05\* | 1.31(0.70-1.92) | 0.71 | 11 | 1.20 (0.90 - 1.50) | 0.41 | 1.11(0.64-1.58) | 0.63 | 1.29(0.79-1.79) | 0.30 |
| | 28 | 23 | 1.09 (0.87 - 1.31) | 0.03\* | 12 | 1.17 (0.79 - 1.55) | 0.13 | 1.12(0.54-1.70) | 0.07 | 1.22(0.57-1.86) | 0.40 | 11 | 1.01 (0.80 - 1.21) | 0.08 | 1.13(0.73-1.54) | >0.99 | 0.88(0.64-1.11) | 0.08 |
Table S2 Comparison of AUCs for SC factors measured by confocal Raman spectrometer at 0,14, and 28 days
In (a) water content, (b) NMF, (c) ceramide, (d) cholesterol, and (e) lactate acid, mean and 95% CI of AUC were calculated for 0-20, 0-12, and 12-20 μm in patients with and without AD. P-values were calculated by Welch’s t-test at 0 vs 14 days and 0 vs 28 days. *P < 0.05 , ** P < 0.01.
AD, atopic dermatitis; AUC, area under the curve; CI, confidence interval; CM, cream-type moisturizer; NMF, natural moisturizing factor; PJ, petroleum jelly.

## Slide 3
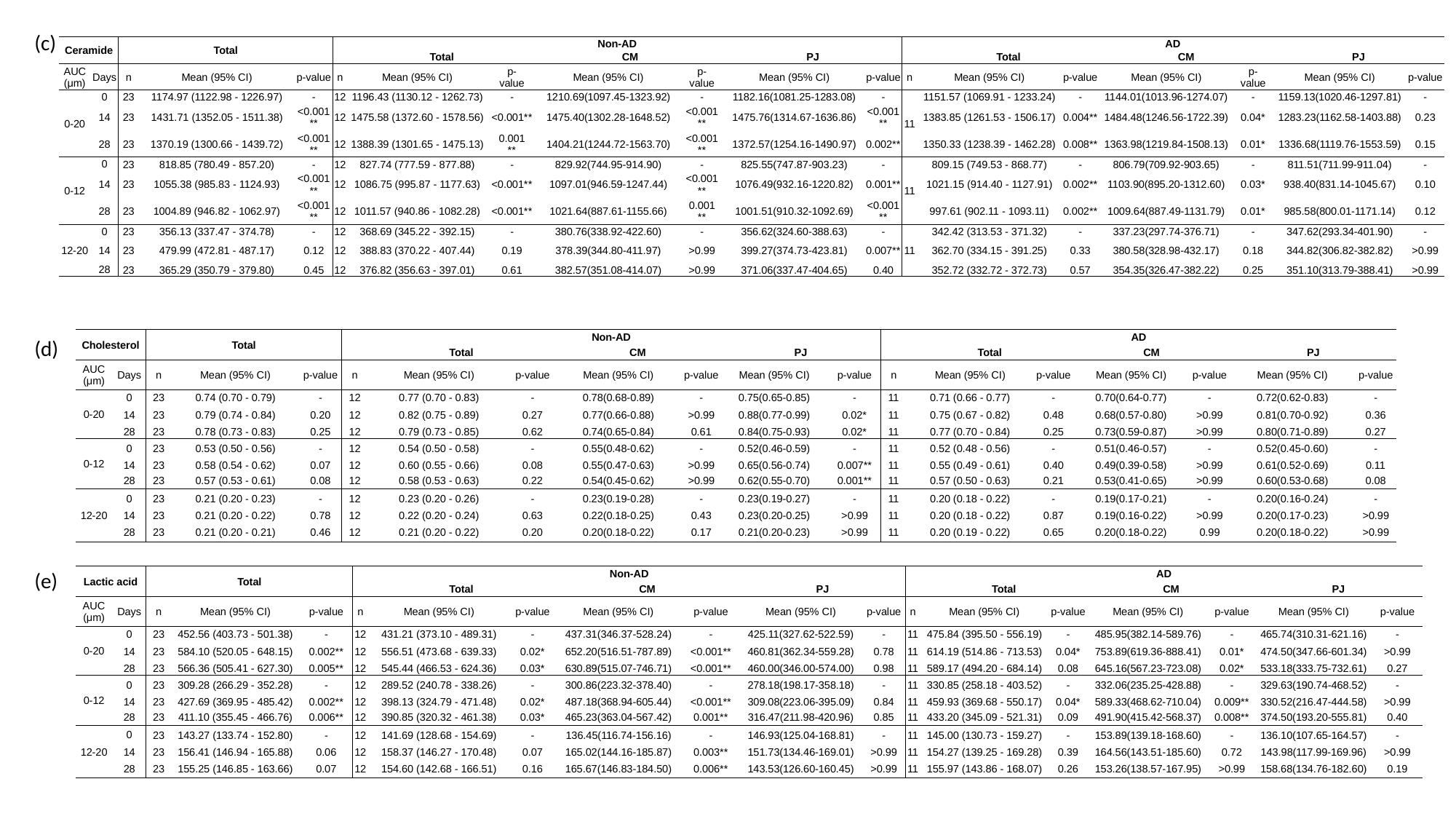

(c)
| Ceramide | | Total | | | Non-AD | | | | | | | AD | | | | | | |
| --- | --- | --- | --- | --- | --- | --- | --- | --- | --- | --- | --- | --- | --- | --- | --- | --- | --- | --- |
| | | | | | | Total | | CM | | PJ | | | Total | | CM | | PJ | |
| AUC (μm) | Days | n | Mean (95% CI) | p-value | n | Mean (95% CI) | p- value | Mean (95% CI) | p- value | Mean (95% CI) | p-value | n | Mean (95% CI) | p-value | Mean (95% CI) | p- value | Mean (95% CI) | p-value |
| 0-20 | 0 | 23 | 1174.97 (1122.98 - 1226.97) | - | 12 | 1196.43 (1130.12 - 1262.73) | - | 1210.69(1097.45-1323.92) | - | 1182.16(1081.25-1283.08) | - | 11 | 1151.57 (1069.91 - 1233.24) | - | 1144.01(1013.96-1274.07) | - | 1159.13(1020.46-1297.81) | - |
| | 14 | 23 | 1431.71 (1352.05 - 1511.38) | <0.001\*\* | 12 | 1475.58 (1372.60 - 1578.56) | <0.001\*\* | 1475.40(1302.28-1648.52) | <0.001 \*\* | 1475.76(1314.67-1636.86) | <0.001\*\* | | 1383.85 (1261.53 - 1506.17) | 0.004\*\* | 1484.48(1246.56-1722.39) | 0.04\* | 1283.23(1162.58-1403.88) | 0.23 |
| | 28 | 23 | 1370.19 (1300.66 - 1439.72) | <0.001\*\* | 12 | 1388.39 (1301.65 - 1475.13) | 0.001 \*\* | 1404.21(1244.72-1563.70) | <0.001 \*\* | 1372.57(1254.16-1490.97) | 0.002\*\* | | 1350.33 (1238.39 - 1462.28) | 0.008\*\* | 1363.98(1219.84-1508.13) | 0.01\* | 1336.68(1119.76-1553.59) | 0.15 |
| 0-12 | 0 | 23 | 818.85 (780.49 - 857.20) | - | 12 | 827.74 (777.59 - 877.88) | - | 829.92(744.95-914.90) | - | 825.55(747.87-903.23) | - | 11 | 809.15 (749.53 - 868.77) | - | 806.79(709.92-903.65) | - | 811.51(711.99-911.04) | - |
| | 14 | 23 | 1055.38 (985.83 - 1124.93) | <0.001\*\* | 12 | 1086.75 (995.87 - 1177.63) | <0.001\*\* | 1097.01(946.59-1247.44) | <0.001 \*\* | 1076.49(932.16-1220.82) | 0.001\*\* | | 1021.15 (914.40 - 1127.91) | 0.002\*\* | 1103.90(895.20-1312.60) | 0.03\* | 938.40(831.14-1045.67) | 0.10 |
| | 28 | 23 | 1004.89 (946.82 - 1062.97) | <0.001\*\* | 12 | 1011.57 (940.86 - 1082.28) | <0.001\*\* | 1021.64(887.61-1155.66) | 0.001 \*\* | 1001.51(910.32-1092.69) | <0.001\*\* | | 997.61 (902.11 - 1093.11) | 0.002\*\* | 1009.64(887.49-1131.79) | 0.01\* | 985.58(800.01-1171.14) | 0.12 |
| 12-20 | 0 | 23 | 356.13 (337.47 - 374.78) | - | 12 | 368.69 (345.22 - 392.15) | - | 380.76(338.92-422.60) | - | 356.62(324.60-388.63) | - | 11 | 342.42 (313.53 - 371.32) | - | 337.23(297.74-376.71) | - | 347.62(293.34-401.90) | - |
| | 14 | 23 | 479.99 (472.81 - 487.17) | 0.12 | 12 | 388.83 (370.22 - 407.44) | 0.19 | 378.39(344.80-411.97) | >0.99 | 399.27(374.73-423.81) | 0.007\*\* | | 362.70 (334.15 - 391.25) | 0.33 | 380.58(328.98-432.17) | 0.18 | 344.82(306.82-382.82) | >0.99 |
| | 28 | 23 | 365.29 (350.79 - 379.80) | 0.45 | 12 | 376.82 (356.63 - 397.01) | 0.61 | 382.57(351.08-414.07) | >0.99 | 371.06(337.47-404.65) | 0.40 | | 352.72 (332.72 - 372.73) | 0.57 | 354.35(326.47-382.22) | 0.25 | 351.10(313.79-388.41) | >0.99 |
(d)
| Cholesterol | | Total | | | Non-AD | | | | | | | AD | | | | | | |
| --- | --- | --- | --- | --- | --- | --- | --- | --- | --- | --- | --- | --- | --- | --- | --- | --- | --- | --- |
| | | | | | | Total | | CM | | PJ | | | Total | | CM | | PJ | |
| AUC (μm) | Days | n | Mean (95% CI) | p-value | n | Mean (95% CI) | p-value | Mean (95% CI) | p-value | Mean (95% CI) | p-value | n | Mean (95% CI) | p-value | Mean (95% CI) | p-value | Mean (95% CI) | p-value |
| 0-20 | 0 | 23 | 0.74 (0.70 - 0.79) | - | 12 | 0.77 (0.70 - 0.83) | - | 0.78(0.68-0.89) | - | 0.75(0.65-0.85) | - | 11 | 0.71 (0.66 - 0.77) | - | 0.70(0.64-0.77) | - | 0.72(0.62-0.83) | - |
| | 14 | 23 | 0.79 (0.74 - 0.84) | 0.20 | 12 | 0.82 (0.75 - 0.89) | 0.27 | 0.77(0.66-0.88) | >0.99 | 0.88(0.77-0.99) | 0.02\* | 11 | 0.75 (0.67 - 0.82) | 0.48 | 0.68(0.57-0.80) | >0.99 | 0.81(0.70-0.92) | 0.36 |
| | 28 | 23 | 0.78 (0.73 - 0.83) | 0.25 | 12 | 0.79 (0.73 - 0.85) | 0.62 | 0.74(0.65-0.84) | 0.61 | 0.84(0.75-0.93) | 0.02\* | 11 | 0.77 (0.70 - 0.84) | 0.25 | 0.73(0.59-0.87) | >0.99 | 0.80(0.71-0.89) | 0.27 |
| 0-12 | 0 | 23 | 0.53 (0.50 - 0.56) | - | 12 | 0.54 (0.50 - 0.58) | - | 0.55(0.48-0.62) | - | 0.52(0.46-0.59) | - | 11 | 0.52 (0.48 - 0.56) | - | 0.51(0.46-0.57) | - | 0.52(0.45-0.60) | - |
| | 14 | 23 | 0.58 (0.54 - 0.62) | 0.07 | 12 | 0.60 (0.55 - 0.66) | 0.08 | 0.55(0.47-0.63) | >0.99 | 0.65(0.56-0.74) | 0.007\*\* | 11 | 0.55 (0.49 - 0.61) | 0.40 | 0.49(0.39-0.58) | >0.99 | 0.61(0.52-0.69) | 0.11 |
| | 28 | 23 | 0.57 (0.53 - 0.61) | 0.08 | 12 | 0.58 (0.53 - 0.63) | 0.22 | 0.54(0.45-0.62) | >0.99 | 0.62(0.55-0.70) | 0.001\*\* | 11 | 0.57 (0.50 - 0.63) | 0.21 | 0.53(0.41-0.65) | >0.99 | 0.60(0.53-0.68) | 0.08 |
| 12-20 | 0 | 23 | 0.21 (0.20 - 0.23) | - | 12 | 0.23 (0.20 - 0.26) | - | 0.23(0.19-0.28) | - | 0.23(0.19-0.27) | - | 11 | 0.20 (0.18 - 0.22) | - | 0.19(0.17-0.21) | - | 0.20(0.16-0.24) | - |
| | 14 | 23 | 0.21 (0.20 - 0.22) | 0.78 | 12 | 0.22 (0.20 - 0.24) | 0.63 | 0.22(0.18-0.25) | 0.43 | 0.23(0.20-0.25) | >0.99 | 11 | 0.20 (0.18 - 0.22) | 0.87 | 0.19(0.16-0.22) | >0.99 | 0.20(0.17-0.23) | >0.99 |
| | 28 | 23 | 0.21 (0.20 - 0.21) | 0.46 | 12 | 0.21 (0.20 - 0.22) | 0.20 | 0.20(0.18-0.22) | 0.17 | 0.21(0.20-0.23) | >0.99 | 11 | 0.20 (0.19 - 0.22) | 0.65 | 0.20(0.18-0.22) | 0.99 | 0.20(0.18-0.22) | >0.99 |
(e)
| Lactic acid | | Total | | | Non-AD | | | | | | | AD | | | | | | |
| --- | --- | --- | --- | --- | --- | --- | --- | --- | --- | --- | --- | --- | --- | --- | --- | --- | --- | --- |
| | | | | | | Total | | CM | | PJ | | | Total | | CM | | PJ | |
| AUC (μm) | Days | n | Mean (95% CI) | p-value | n | Mean (95% CI) | p-value | Mean (95% CI) | p-value | Mean (95% CI) | p-value | n | Mean (95% CI) | p-value | Mean (95% CI) | p-value | Mean (95% CI) | p-value |
| 0-20 | 0 | 23 | 452.56 (403.73 - 501.38) | - | 12 | 431.21 (373.10 - 489.31) | - | 437.31(346.37-528.24) | - | 425.11(327.62-522.59) | - | 11 | 475.84 (395.50 - 556.19) | - | 485.95(382.14-589.76) | - | 465.74(310.31-621.16) | - |
| | 14 | 23 | 584.10 (520.05 - 648.15) | 0.002\*\* | 12 | 556.51 (473.68 - 639.33) | 0.02\* | 652.20(516.51-787.89) | <0.001\*\* | 460.81(362.34-559.28) | 0.78 | 11 | 614.19 (514.86 - 713.53) | 0.04\* | 753.89(619.36-888.41) | 0.01\* | 474.50(347.66-601.34) | >0.99 |
| | 28 | 23 | 566.36 (505.41 - 627.30) | 0.005\*\* | 12 | 545.44 (466.53 - 624.36) | 0.03\* | 630.89(515.07-746.71) | <0.001\*\* | 460.00(346.00-574.00) | 0.98 | 11 | 589.17 (494.20 - 684.14) | 0.08 | 645.16(567.23-723.08) | 0.02\* | 533.18(333.75-732.61) | 0.27 |
| 0-12 | 0 | 23 | 309.28 (266.29 - 352.28) | - | 12 | 289.52 (240.78 - 338.26) | - | 300.86(223.32-378.40) | - | 278.18(198.17-358.18) | - | 11 | 330.85 (258.18 - 403.52) | - | 332.06(235.25-428.88) | - | 329.63(190.74-468.52) | - |
| | 14 | 23 | 427.69 (369.95 - 485.42) | 0.002\*\* | 12 | 398.13 (324.79 - 471.48) | 0.02\* | 487.18(368.94-605.44) | <0.001\*\* | 309.08(223.06-395.09) | 0.84 | 11 | 459.93 (369.68 - 550.17) | 0.04\* | 589.33(468.62-710.04) | 0.009\*\* | 330.52(216.47-444.58) | >0.99 |
| | 28 | 23 | 411.10 (355.45 - 466.76) | 0.006\*\* | 12 | 390.85 (320.32 - 461.38) | 0.03\* | 465.23(363.04-567.42) | 0.001\*\* | 316.47(211.98-420.96) | 0.85 | 11 | 433.20 (345.09 - 521.31) | 0.09 | 491.90(415.42-568.37) | 0.008\*\* | 374.50(193.20-555.81) | 0.40 |
| 12-20 | 0 | 23 | 143.27 (133.74 - 152.80) | - | 12 | 141.69 (128.68 - 154.69) | - | 136.45(116.74-156.16) | - | 146.93(125.04-168.81) | - | 11 | 145.00 (130.73 - 159.27) | - | 153.89(139.18-168.60) | - | 136.10(107.65-164.57) | - |
| | 14 | 23 | 156.41 (146.94 - 165.88) | 0.06 | 12 | 158.37 (146.27 - 170.48) | 0.07 | 165.02(144.16-185.87) | 0.003\*\* | 151.73(134.46-169.01) | >0.99 | 11 | 154.27 (139.25 - 169.28) | 0.39 | 164.56(143.51-185.60) | 0.72 | 143.98(117.99-169.96) | >0.99 |
| | 28 | 23 | 155.25 (146.85 - 163.66) | 0.07 | 12 | 154.60 (142.68 - 166.51) | 0.16 | 165.67(146.83-184.50) | 0.006\*\* | 143.53(126.60-160.45) | >0.99 | 11 | 155.97 (143.86 - 168.07) | 0.26 | 153.26(138.57-167.95) | >0.99 | 158.68(134.76-182.60) | 0.19 |

## Slide 4
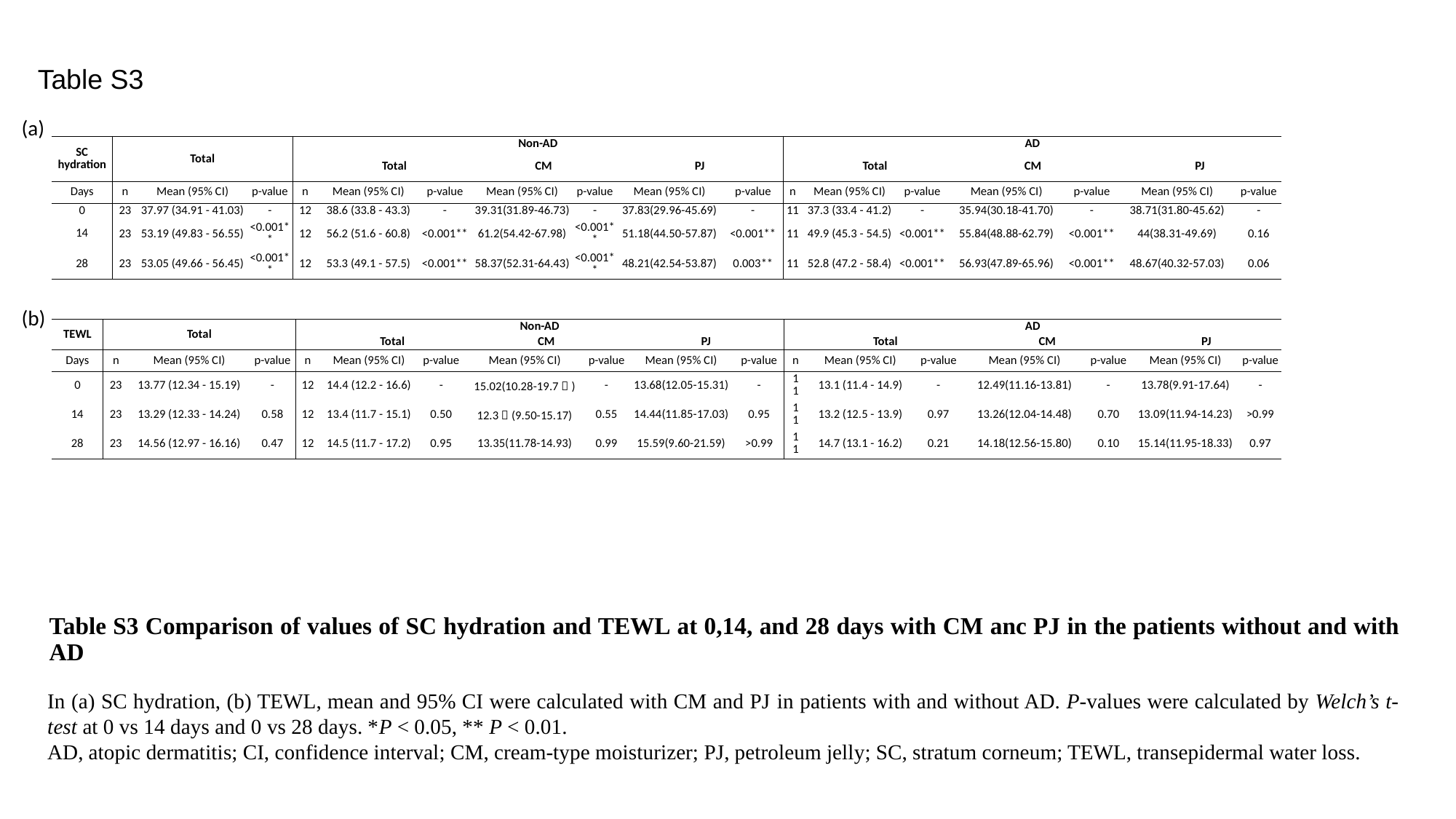

Table S3
(a)
| SC hydration | Total | | | Non-AD | | | | | | | AD | | | | | | |
| --- | --- | --- | --- | --- | --- | --- | --- | --- | --- | --- | --- | --- | --- | --- | --- | --- | --- |
| | | | | | Total | | CM | | PJ | | | Total | | CM | | PJ | |
| Days | n | Mean (95% CI) | p-value | n | Mean (95% CI) | p-value | Mean (95% CI) | p-value | Mean (95% CI) | p-value | n | Mean (95% CI) | p-value | Mean (95% CI) | p-value | Mean (95% CI) | p-value |
| 0 | 23 | 37.97 (34.91 - 41.03) | - | 12 | 38.6 (33.8 - 43.3) | - | 39.31(31.89-46.73) | - | 37.83(29.96-45.69) | - | 11 | 37.3 (33.4 - 41.2) | - | 35.94(30.18-41.70) | - | 38.71(31.80-45.62) | - |
| 14 | 23 | 53.19 (49.83 - 56.55) | <0.001\*\* | 12 | 56.2 (51.6 - 60.8) | <0.001\*\* | 61.2(54.42-67.98) | <0.001\*\* | 51.18(44.50-57.87) | <0.001\*\* | 11 | 49.9 (45.3 - 54.5) | <0.001\*\* | 55.84(48.88-62.79) | <0.001\*\* | 44(38.31-49.69) | 0.16 |
| 28 | 23 | 53.05 (49.66 - 56.45) | <0.001\*\* | 12 | 53.3 (49.1 - 57.5) | <0.001\*\* | 58.37(52.31-64.43) | <0.001\*\* | 48.21(42.54-53.87) | 0.003\*\* | 11 | 52.8 (47.2 - 58.4) | <0.001\*\* | 56.93(47.89-65.96) | <0.001\*\* | 48.67(40.32-57.03) | 0.06 |
(b)
| TEWL | Total | | | Non-AD | | | | | | | AD | | | | | | |
| --- | --- | --- | --- | --- | --- | --- | --- | --- | --- | --- | --- | --- | --- | --- | --- | --- | --- |
| | | | | | Total | | CM | | PJ | | | Total | | CM | | PJ | |
| Days | n | Mean (95% CI) | p-value | n | Mean (95% CI) | p-value | Mean (95% CI) | p-value | Mean (95% CI) | p-value | n | Mean (95% CI) | p-value | Mean (95% CI) | p-value | Mean (95% CI) | p-value |
| 0 | 23 | 13.77 (12.34 - 15.19) | - | 12 | 14.4 (12.2 - 16.6) | - | 15.02(10.28-19.7７) | - | 13.68(12.05-15.31) | - | 11 | 13.1 (11.4 - 14.9) | - | 12.49(11.16-13.81) | - | 13.78(9.91-17.64) | - |
| 14 | 23 | 13.29 (12.33 - 14.24) | 0.58 | 12 | 13.4 (11.7 - 15.1) | 0.50 | 12.3４(9.50-15.17) | 0.55 | 14.44(11.85-17.03) | 0.95 | 11 | 13.2 (12.5 - 13.9) | 0.97 | 13.26(12.04-14.48) | 0.70 | 13.09(11.94-14.23) | >0.99 |
| 28 | 23 | 14.56 (12.97 - 16.16) | 0.47 | 12 | 14.5 (11.7 - 17.2) | 0.95 | 13.35(11.78-14.93) | 0.99 | 15.59(9.60-21.59) | >0.99 | 11 | 14.7 (13.1 - 16.2) | 0.21 | 14.18(12.56-15.80) | 0.10 | 15.14(11.95-18.33) | 0.97 |
Table S3 Comparison of values of SC hydration and TEWL at 0,14, and 28 days with CM anc PJ in the patients without and with AD
In (a) SC hydration, (b) TEWL, mean and 95% CI were calculated with CM and PJ in patients with and without AD. P-values were calculated by Welch’s t-test at 0 vs 14 days and 0 vs 28 days. *P < 0.05, ** P < 0.01.
AD, atopic dermatitis; CI, confidence interval; CM, cream-type moisturizer; PJ, petroleum jelly; SC, stratum corneum; TEWL, transepidermal water loss.

## Slide 5
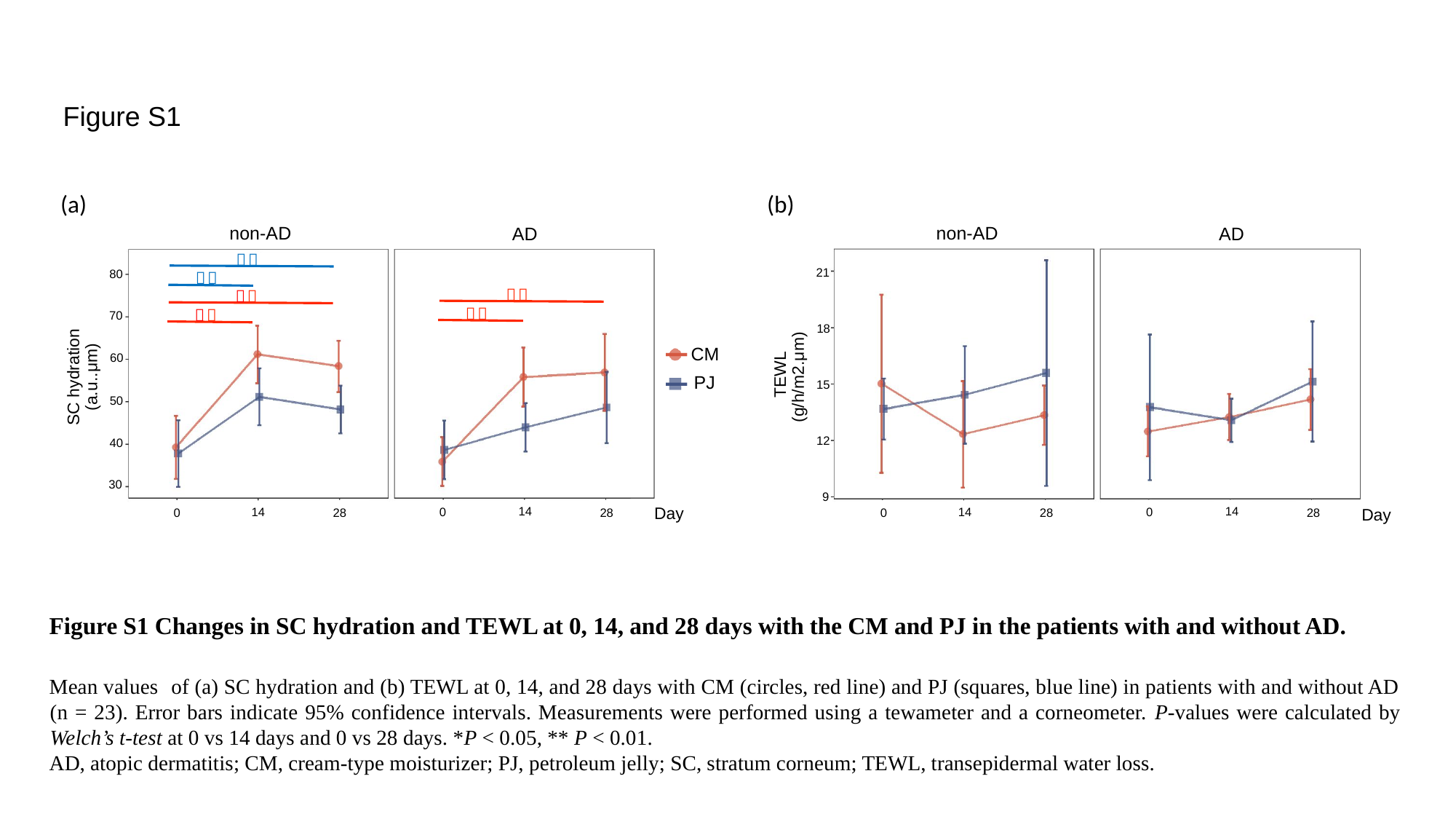

Figure S1
(a)
(b)
non-AD
AD
non-AD
AD
＊
＊
＊
＊
21
18
15
12
 9
TEWL (g/h/m2.μm)
0
14
28
28
0
14
80
70
60
50
40
30
SC hydration
(a.u..μm)
0
14
28
28
0
14
＊
＊
＊
＊
＊
＊
＊
＊
CM
PJ
Day
Day
Figure S1 Changes in SC hydration and TEWL at 0, 14, and 28 days with the CM and PJ in the patients with and without AD.
Mean values of (a) SC hydration and (b) TEWL at 0, 14, and 28 days with CM (circles, red line) and PJ (squares, blue line) in patients with and without AD (n = 23). Error bars indicate 95% confidence intervals. Measurements were performed using a tewameter and a corneometer. P-values were calculated by Welch’s t-test at 0 vs 14 days and 0 vs 28 days. *P < 0.05, ** P < 0.01.
AD, atopic dermatitis; CM, cream-type moisturizer; PJ, petroleum jelly; SC, stratum corneum; TEWL, transepidermal water loss.
